# MAYA (Multiple ActivitY Analyzer): An Open Access Tool to Explore Structure‐Multiple Activity Relationships in the Chemical Universe

**DOI:** 10.1002/minf.202400306

**Published:** 2025-02-11

**Authors:** J. Israel Espinoza‐Castañeda, José L. Medina‐Franco

**Affiliations:** ^1^ J. Israel Espinoza-Castañeda - DIFACQUIM Research Group Department of Pharmacy School of Chemistry Universidad Nacional Autónoma de México Avenida Universidad 3000 Mexico City 04510 Mexico; ^2^ José L. Medina-Franco - DIFACQUIM Research Group Department of Pharmacy School of Chemistry Universidad Nacional Autónoma de México Avenida Universidad 3000 Mexico City 04510 Mexico

**Keywords:** automatic data visualization, chemical space, chemical multiverse, chemoinformatics, open science, structure-multiple activity relationships (SMARts)

## Abstract

Herein, we introduce MAYA (Multiple Activity Analyzer), a tool designed to automatically construct a chemical multiverse, generating multiple visualizations of chemical spaces of a compound data set described by structural descriptors of different nature such as Molecular ACCess Systems (MACCS) keys, extended connectivity fingerprints with different radius, molecular descriptors with pharmaceutical relevance, and bioactivity descriptors. These representations are integrated with various data visualization techniques for the automated analysis focused on structure ‐ multiple activity/property relationships, enabling analysis for various problems set in user‐friendly source software. The source code of MAYA is freely available on GitHub at https://github.com/IsrC11/MAYA.git.

## Graphical abstract

MAYA is an open chemical multiverse visualizer tool for the automated exploration of structure‐multiple activity relationships (SMARts).

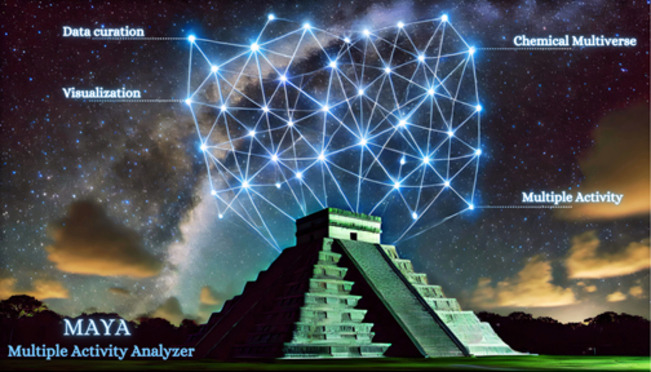

## 1 Introduction

Analyzing structure‐activity relationships (SAR) is a fundamental concept in medicinal chemistry and a key activity in drug discovery and development [1, 2]. SAR approach applied to multiparametric datasets is crucial to extract information about chemical modifications and their impact on physicochemical properties or activity. This information offers a practical approach for rational optimization in compound design, providing an improved strategy for drug discovery. By correlating molecular information not only with biological activity but also with other properties such as electronic characteristics, predicted scores or drug‐likeness descriptors, it becomes possible to optimize multiple physicochemical and biological attributes, this optimizations can enhance potency, reduce toxicity or ensure bioavailability. Likewise, exploring structure‐multiple activity relationships (SMARts) is becoming a standard practice in drug discovery due to the increased awareness of polypharmacology and the well‐known fact that active bioactive compounds interact not only with desired molecular targets but also with off‐targets, leading to undesirable side effects [3, 4].

A systematic manner to explore SARs and SMARts qualitatively and quantitatively is through the analysis of chemical space, also called chemical universe, a fundamental concept in chemoinformatics [5, 6, 7]. The chemical space has been defined as a multi‐dimensional descriptor space where the compounds can be located [8]. Adding one or more biological activities to the chemical space gives rise to a so‐called chemogenomics space [9], enabling the qualitative exploration or systematic and quantitative analysis of SARs or SMARts [10, 11]. Since the chemical space of a set of compounds strongly depends on the set of descriptors to define the multi‐dimensional space, it has been suggested that multiple descriptors describe better compound data sets [12], giving rise to the concept of chemical multiverse [13].

In many practical SARs or SMARts analysis under the chemical space framework, data visualization techniques are used. To this end, several methods have been developed and implemented [14, 15, 16, 17, 18, 19]. In favor of open access tools and democratization of science [20], there is a need to develop open access tools to automate the generation of visual representations of chemical space and chemical multiverse of compound data sets to contribute to the analysis of SARs or SMARts.

This manuscript aims to introduce and describe MAYA: an open‐access tool to explore the chemical universe and multiverse of compound data sets annotated with multiple biological endpoints. The tool is general and can be adapted and extended to include various molecular descriptors (e. g., fingerprints), properties and several calculated scores other than biological activities. The tool can be used in various applications such as multi‐target drug design, analysis of on and off‐target effects, and analysis of virtual screening results where a given set of compounds are annotated with calculated scores. Herein, we illustrate the application of MAYA to analyze SMARts of an exemplary set of compounds of therapeutic relevance.

## 2 Methods

**Figure 1:**
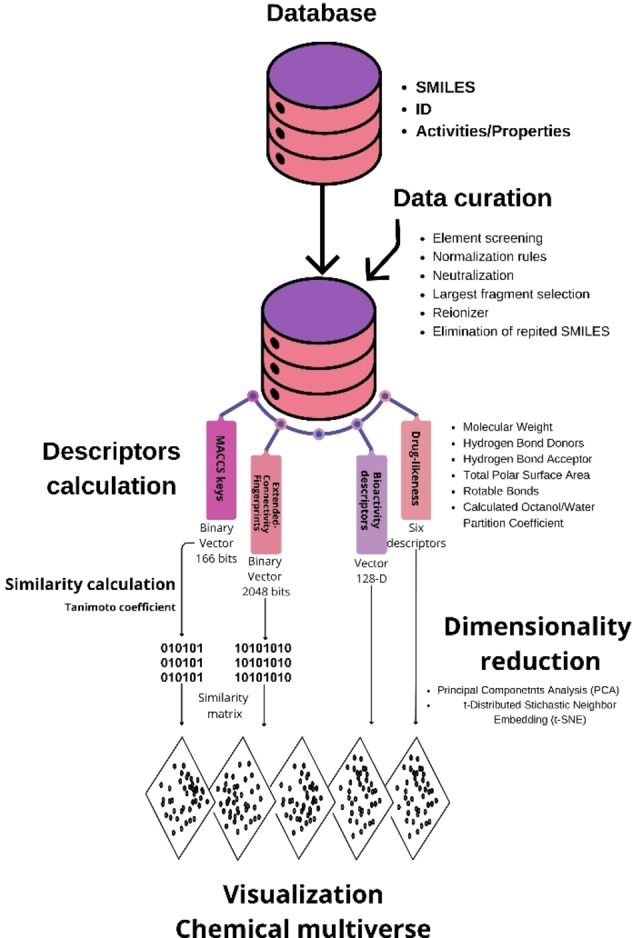
MAYA′s general workflow and main steps include the user′s input of chemical structures with multiple activities, data curation, descriptor and similarity calculations, and data visualization using dimensionality reduction.

The general overview of MAYA is outlined in Figure 1. The tool performs data curation and standardization for a given data set of user‐supplied compounds represented as simplified molecular input line entry system (SMILES) strings [21] annotated with bioactivity data. Then, MAYA computes descriptors, performs pairwise similarity calculations, and reduces dimensionality to generate visual representations of the chemical space based on the different descriptors. Details of each step are described below. For the validation of the visualizations, the metrics Trustworthiness, Continuity and Correlation are integrated to evaluate the quality and consistency of the visualization. A detailed description can be found in the user guide: https://github.com/IsrC11/MAYA/blob/main/User_Guide.md.

### 2.1 Data Curation

MAYA has implemented a data curation protocol from MolVS [22] that performs the following operations:

1. Screening of Allowed Elements: Limits the analysis to elements applicable within the current drug discovery domain.

2. Standardization: Ensures consistent molecular representations, generating canonical SMILES through these operations:

a. Normalization rules: Corrects structural inconsistencies, transforms functional groups to their correct forms and adjusts charge as necessary. A list of reaction SMARTS (SMILES Arbitrary Target Specifications) patterns is applied iteratively to each molecule′s SMILES until no further modifications occurs or a maximum iterations limit is reached. This includes patterns such as:

i. Separating charges in sulfones, pyridine oxides, etc.

ii. Adjusting charges in azides, diazo and azo groups.

iii. Resolving hydrazine‐diazonium systems.

b. Neutralization (Uncharge): Adjusts the molecule′s hydrogen count to archive a neutral state.

c. Largest Fragment Selector: identifies the main covalent fragment in molecule that contain multiple disconnected components.

d. Reionizer: Applied SMARTS patterns to pinpoint and swap protons between the strongest protonated acid and the weakness ionized acid in the molecule. This operation is repeated as needed to archive the proper ionic or neutral form.

e. Canonical Tautomer Selection: Applies predefined SMARTS patterns to adjust hydrogen positioning and bond type, alongside a scoring system that ranks substructures to establish the canonical tautomer.

f. Elimination of repeated SMILES.

### 2.2 Descriptors

The current set of descriptors implemented in the last version of MAYA are Molecular ACCes System (MACCS) keys (166‐bits) [23], extended connectivity fingerprints (ECFP) of diameter 4 and 6 (ECFP4, ECFP6) (2048‐bits) [24]; six continuous properties of pharmaceutical relevance, and bioactivity descriptors. The properties of pharmaceutical relevance are molecular weight (MW), calculated octanol/water partition coefficient (cLogP), topological polar surface area (TPSA), number of hydrogen bond donors (HBD), number of hydrogen bond acceptors (HBA), and number of rotatable bonds (RB), both fingerprints and molecular descriptors are calculated using the RDkit library. The bioactivity descriptors offer a new opportunity to integrate bioactivity knowledge into similarity calculations, including for compounds that are not annotated with experimental data. Bioactivity descriptors are organized into five levels of complexity; A: Chemistry, B: Targets, C: Networks, D: Cells and E: Clinical, each further divided into five sublevels, resulting in 25 distinct spaces. These signatures provides an innovative alternative for characterizing compounds. For each of 25 spaces, a Siamese Neural Network (SNN) is trained using available experimental data or simulates missing information. Each SNN produces a 128‐dimensional vector, where each dimension represents an abstract learned feature optimized for assessing molecular similarities, rather than direct chemical properties. Further details of the biological descriptors included in the current version of MAYA are described elsewhere [25].

### 2.3 Similarity Calculations, Dimensionality Reduction, and Visualization of Chemical Space

The pairwise similarity calculations are performed with the Tanimoto coefficient [26] applied to MACCS keys and ECFP 4 or 6. For the generation of chemical spaces, the similarity matrix of structural descriptors is used. In the case of chemical spaces derived from bioactivity and drug‐likeness descriptors, the resulting vector and numerical values are directly employed. The data visualization techniques to obtain visual representation of the chemical space implemented in the current version of MAYA are principal component analysis (PCA) [27] and t‐distributed stochastic neighbor embedding (t‐SNE) through the scikit‐learn library [28]. MAYA includes as default an “mpIC_50_” metric which represents the mean of the activity values of each compound across the different biological endpoints. The mpIC_50_ can be mapped on the visualizations of the chemical spaces using a continuous color scale.

### 2.4 Visualizations

The generated visualizations are interactive, facilitating compound identification. These include a pop‐up window that displays the compound ID, SMILES, properties and a 2D representation. Additionally, the visualizations feature a color scale that, by default, represents the mean pIC50 (referred to as mpIC50) and point sizes based on another property, which defaults to the standard deviation of the activities. However, users can customize these features, such as changing the displayed property or disabling them as needed.

The mpIC50 is calculated as the average of the values reported for each compound′s activities. Meanwhile, the standard deviation is computed based on these activities and normalized to a range of 0 to 1, allowing the adjustment of the minimum point size in the plot and visualizing compounds with low or negligible standard deviation.

MAYA is designed with a user‐friendly configuration, enhancing users to configure the displayed properties and customize the color palettes through the command line. The visualization color palette can be customized through the ‘palette’ variable in the execution line, this allows users to specify predefined palette names from the Matplotlib library or input list color codes.

A more detailed explanation of this implementations are in the User′s Guide: https://github.com/IsrC11/MAYA/blob/main/User_Guide.md.

### 2.5 Comparison with other Tools

We compared the main features of MAYA with other software tools such as Activity Landscape Plotter [29], which implement DAD (Dual Activity Difference) and TAD (Triple Activity Difference) maps, as well as SARANEA [30].

DAD/TAD maps are visualizations that represent activity differences between pairs for two or three targets. These maps incorporate pairwise similarity calculations across multiple descriptors, generating a consensus for SAR characterization. They enable the exploration of single, dual or triple activity cliff and scaffold hops. Conversely, while MAYA does not directly integrate activity cliff analysis in its code, this information can be represented within a chemical based on similarity values and using the color scale to indicate activity values against a target. However, the efficient implementation of quantitative analysis of dual or triple activity cliffs is not currently available, although their inclusion could be valuable in future developments. Additionally, MAYA supports detailed analysis of individual descriptors and integration of a large number of targets.

SARANEA, on the other hand, is a free application development for interactive analysis of SAR and structure‐selectivity relationships (SSR). Among its main features is the generation of network‐like similarity graphs to analyze the potency or selectivity of compounds against multiple targets, based on a single descriptor. It also generates a cliff index and SAR trees to explore activity or selectivity. In an applied study, SARANEA has demonstrated its ability to identify key structural features influencing compound potency and selectivity across multiple targets, facilitating compound optimization. In contrast, MAYA stands out for its focus on interactive visualizations using multiple descriptors and simultaneous analysis of multiple activities or properties, making it ideal for broad exploration studies. Conversely, tools like SARANEA and DAD/TAD maps are more suitable for specific SAR and SSR analysis.

### 2.6 Current Limitations and Workaround

MAYA needs to perform multiple pairwise similarity calculations, which can result in prolonged computational times when handing databases containing thousands or more. To optimize this process, similarity calculations and data curation has been designed to run in parallel.

For the local version, it is recommended to work with databases of up to 50000 compounds, considering the scalability if computation times. For instance, visualizing a database of 2000 compounds can be archived approximately 30 minutes. In the MAYA′s version available on Google Colaboratory, it is advisable to use databases containing up to 2000 compounds. This recommendation is due to the limitations of the platform, which provides 12 GB of RAM, a runtime of 12 hours per session and 2 CPU cores. In this environment, computation times can reach approximately 75 minutes. However, this will depend on the available computational resources and the specific calculation required by the user. (A comparative table of estimated computation times for different database sizes, using both local and Google Colaboratory configurations are presented in Table S1 is the Supporting Information).

## 3 Results and Discussion

MAYA is an automated application for chemical multiverse visualization since it enables the fast generation of chemical spaces of the same data set using different types of descriptors. Since the tool also enables the fast mapping of multiple biological endpoints (e. g., multiple activities), the application generates a group of chemical spaces of the set of chemical compounds represented with multiple descriptors and portraying multiple biological endpoints (Figure 1).

To illustrate the application of MAYA, an experimental database comprising approximately 3800 compounds tested against 172 protein kinases [31] was used as the starting point. However, the analysis was restricted to compounds with SMILES notation, resulting in a final dataset of 1497 compounds annotated with pK_I_ values for the 172 protein kinases and a promiscuity score. Given that pK_I_ values were inconsistent across the 172 protein kinases with missing values or, in some cases, only a maximum value reported compounds were classified into four categories:

- Active: compounds with a reported pKI value equal to or greater than 6, labeled with a score of 4.
- Non‐specific: compounds with pKI values greater than or equal to 4.5 but less than 6, labeled with a score of 3.
- Inactive: compounds with pKI values below 4.5, labeled with a score of 2.
- Unknown: compounds without reported pKI values, labeled with a score of 1.

**Figure 2:**
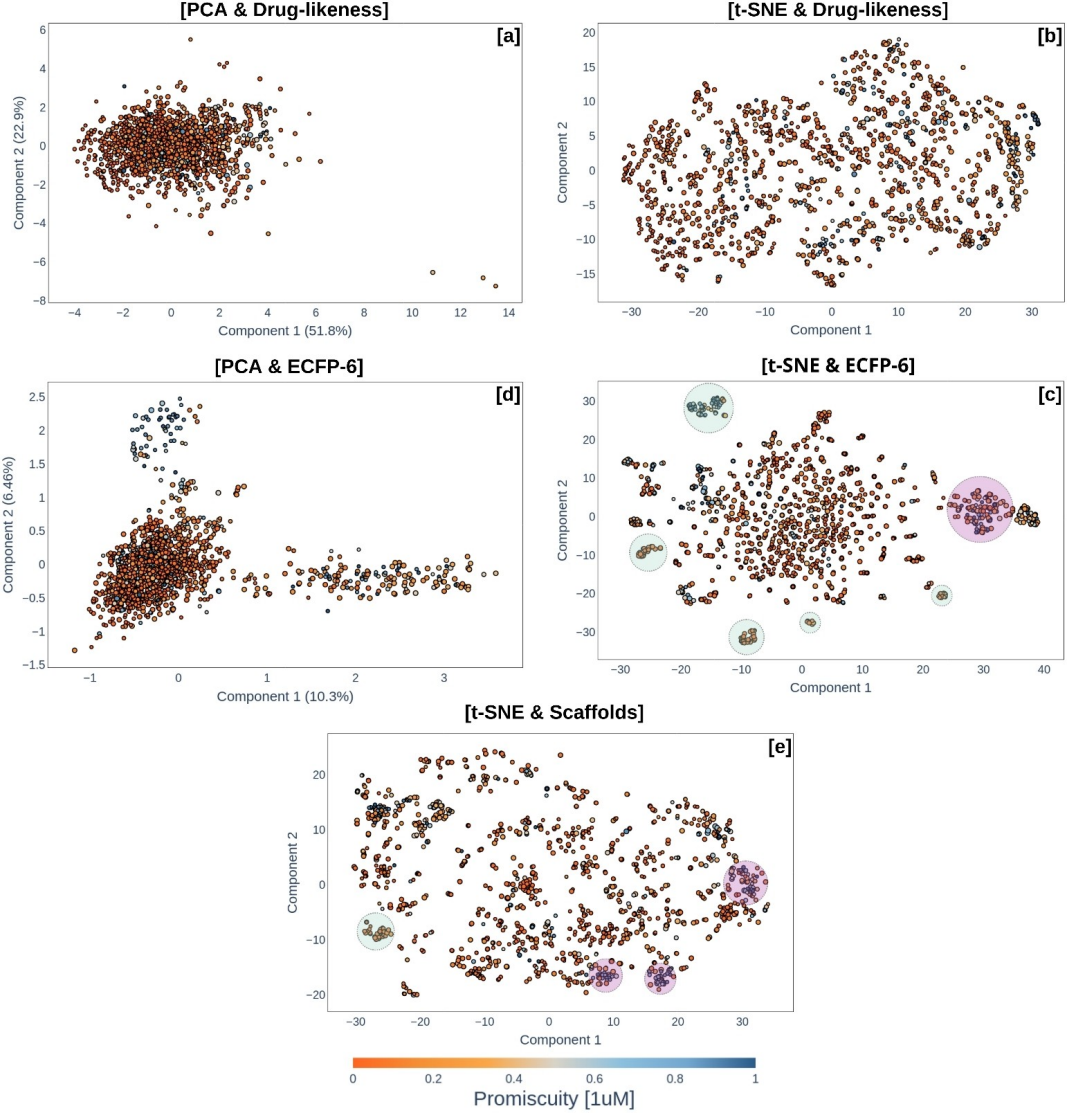
Visual representation of the chemical multiverse automatically generated with MAYA. This analysis includes 1497 compounds annotated with pK_I_ values against 172 kinase proteins and a promiscuity score. The color scale represents the level of promiscuity, where orange tones indicate low values, and the size of the points reflects compound score against the 172 proteins. [a] Chemical space constructed using PCA with six drug‐likeness descriptors (MW, cLogP, TPSA, HBD, HBA, and RB), capturing 74.7% of the total variability (Trustworthiness=0.891 and Correlation=0.940). [b] Visualization generated using t‐SNE with the same drug‐likeness descriptors. The perplexity value for all t‐SNE visualizations was set to 50 (Trustworthiness=0.995 and Correlation=0.578). [c] Representation based on ECFP descriptors with a radius of 3 using t‐SNE (Trustworthiness=0.990 and Correlation=0.734). A global activity cliff region is highlighted in pink, while green regions indicate areas of interest for optimizing multi‐target activity compounds. [d] Chemical space generated with drug‐likeness descriptors using PCA (Trustworthiness=0.785 and Correlation=0.802). Here, the variability retained was low, with only 16.76% explained by the first two principal components. [e] Space constructed with bioactivity descriptors based on scaffolds (Trustworthiness=0.994 and Correlation=0.50). Pink regions highlight clusters of compounds with similar scaffolds and high promiscuity values, while green regions indicate clusters with low promiscuity scaffolds.

Finally, a score was generated for the 1492 compounds by summing their ranking values with respect to the 172 protein kinases. The results obtained using MAYA are presented in Figure 2, showcasing six representative visualizations of the chemical space (additional visualizations are available in Figure S2 of the Supplementary Information). These visualizations include representations of chemical space using t‐SNE and PCA with three types of descriptors: ECFP6, continuous drug‐likeness properties, and bioactivity descriptors. In the presented visualizations, the color scale from orange to blue represents the promiscuity score, defined as the fraction of compounds inhibiting a kinase at 1 μM or better versus 100 nM or better. The size of the points corresponds to the global classification score, with larger points indicating higher activity against the 172 protein kinases.

The chemical space in Figure 2a shows that the compounds do not exhibit significant differences in drug‐likeness properties across the 172 kinase proteins, as the most active compounds (represented by larger points) and the least active compounds share the same chemical space. Therefore, it can be concluded that selectivity optimizations aimed at improving any of the included properties are not relevant, since both active and inactive, as well as promiscuous and non‐promiscuous compounds, are highly similar with respect to the six druglikeness descriptors. Three points with low promiscuity scores stand out as outliers. On the other hand, using t‐SNE (Figure 2b) provides a better distribution of the data, enabling the analysis of local regions.

Chemical space Figure 2c, constructed with the topological descriptor ECFP radius 3 and t‐SNE, exhibits a greater degree of dispersion compared to space 2a, due to structural diversity. However, local clusters of compounds with a promiscuity value (orange shades) are observed, highlighting areas of interest for guiding the development of selective and active compounds for multiple targets. Additionally, it is possible to identify regions of global activity cliffs by observing areas of chemical space with a mixture of data points, representing a blend of structurally similar compounds that show significantly different activity for the 172 kinase proteins. In contrast, for the chemical space constructed with PCA (Figure 2d), the recovery of the initial data variability for the first two principal components is only 16.76%.

Among the bioactivity descriptors, the one related to scaffold comparison is included. As a result, in chemical space (Figure 2e), regions corresponding to compounds with high promiscuity across the 172 kinase proteins are identified. These results have the potential to guide optimization by discarding scaffolds with high similarity to promiscuous compounds and prioritizing those with low promiscuity and high activity.

Regarding the validation of the visualizations, Trustworthiness values above 0.9 and correlation values above 0.7 were obtained for most chemical spaces, confirming that the projections in the reduced two‐dimensional space preserve the relationships of compounds in the original space, with similar compounds projected close to each other. However, the chemical spaces generated using t‐SNE & Druglikeness and t‐SNE & Scaffolds did not maintain similarity relationships, showing a Correlation values of 0.578 and 0.5 respectively.

These results highlight the importance of integrating different types of descriptors and visualization techniques to enhance the characterization and analysis of chemical space. The computation time was approximately 70 minutes.

## 4 Conclusions

MAYA is an open‐access tool to analyze the chemical space and chemical multiverse of compound data sets annotated with multiple biological activities. With MAYA, it is possible to customize the visual representation of the chemical space by modifying the color palette, transparency, size, and shape of each data point. The visualizations generated are interactive graphs useful for in‐depth analysis of each data point, allowing for the retrieval of SMILES codes, visualization of the chemical structures, and an activity index value, as a measure of the compound′s multi‐activity profile. A straightforward application of the free tool is exploring SMARts of compound data sets. The tool is general and can be extended to analyze properties other than biological activities. The code of MAYA is freely available at https://github.com/IsrC11/MAYA.git.

Future developments in MAYA include adding other types of (structural or biological) descriptors and implementing similarity indices other than the Tanimoto coefficient. Similarly, in addition to PCA and t‐SNE, other data visualization techniques can be incorporated. Also, MAYA can be adapted to analyze other types of properties. For example, data exploration of molecules annotated with different flavors or aromas (e. g., different measured properties), compounds data sets with multiple toxicity profiles (applications for pesticides), etc., making MAYA a flexible and free‐access expandable resource for structure multiple‐property relationship analysis.

## Supporting Information

A comparison of estimated computation times for different database sizes (Table S1). Summary of the validation metrics obtained from multiple databases (Table S2). Chemical multiverse of 2309 FDA approved compounds (Figure S1 and S2). Visual representation of the chemical multiverse obtained automatically with MAYA using biological descriptors (Figure S3). Chemical multiverse constructed with MAYA of a dataset of 170 compounds. (Figures S4 and S5).

## Conflict of Interests

The authors declare no conflicts of interest.

5

## Supporting information

As a service to our authors and readers, this journal provides supporting information supplied by the authors. Such materials are peer reviewed and may be re‐organized for online delivery, but are not copy‐edited or typeset. Technical support issues arising from supporting information (other than missing files) should be addressed to the authors.

Supporting Information

## Data Availability

The source code for MAYA is freely available on GitHub: https://github.com/IsrC11/MAYA.git. A step‐by‐step guide for implementation and usage is described in the User_Guide.md file. All the necessary packages are integrated to be installed automatically in Google Colaboratory. The input files for the three targets discussed in the case study can be found on the GitHub repository.
