# Supplementary material for: MAYA (Multiple ActivitY Analyzer): An Open Access Tool to Explore Structure‐Multiple Activity Relationships in the Chemical Universe
Source: Mol Inform. 2025 Feb 11;44(2):e202400306. doi: 10.1002/minf.202400306 (PMC11812492; doi:10.1002/minf.202400306)

Molecular Informatics

Supporting Information

MAYA (Multiple ActivitY Analyzer): An Open Access Tool to Explore Structure-Multiple Activity Relationships in the Chemical Universe

J. Israel Espinoza-Castañeda[1] | José L. Medina-Franco[2]

SUPPORTING INFORMATION

MAYA (Multiple ActivitY Analyzer): An Open Access Tool to Explore Structure-Multiple Activity Relationships in the Chemical Universe

J. Israel Espinoza-Castañeda, José L. Medina-Franco*

DIFACQUIM Research Group, Department of Pharmacy, School of Chemistry, Universidad Nacional Autónoma de México, Avenida Universidad 3000, Mexico City 04510, Mexico

Content

Table S1. Comparative table of computational times for local and Google Colaboratory configurations of MAYA.

Table S2. Summary of the metrics obtained from the analysis of multiple compounds databases of different natures.

Figure S1. Descriptive analysis of the properties of the 2309 FDA approved compounds.

Figure S2. Visualization validation using 2309 FDA (Food and Drug Administration) approved compounds.

Figure S3. Remaining visualizations obtained for database of 1492 compounds against 172 kinases.

Figure S4. Chemical multiverse constructed with MAYA for the analysis of a dataset of 170 compounds annotated with inhibitory activity (IC50) against three biological endpoints, specifically with ChEMBL identifiers: CHEMBL3142, CHEMBL5147 and CHEMBL3234.

Figure S5. Visual representation of the chemical multiverse obtained automatically with MAYA using biological descriptors for the same database used in Figure S3.

Table S1. Comparative table of estimated computation times for each process integrated into MAYA, across different database sizes, using both local and Google Collaboratory configurations.

Database size	Process	Local version (64GB RAM, 10CPU cores) [minutes]	Google Colab (12GB, 2CPU cores) [minutes]
100	5 minutes		5 minutes
1000	Curation process	3	7
	Similariy calculation (MACCS keys)	5	15
	Similariy calculation (ECFP)	3	10
	Calculation drug-likeness descriptors	1	3
	25 bioactivity descriptors calculation	7	10
	**Total**	**19 minutes**	**45 minutes**
2000	Curation process	7	8
	Similariy calculation (MACCS keys)	8	22
	Similariy calculation (ECFP)	7	17
	Calculation drug-likeness descriptors	2	3
	25 bioactivity descriptors calculation	6	25
	**Total**	**30 minutes**	**75 minutes**
5000	Curation process	12	24
	Similariy calculation (MACCS keys)	19	47
	Similariy calculation (ECFP)	16	42
	Calculation drug-likeness descriptors	4	7
	25 bioactivity descriptors calculation	20	45
	**Total**	**71 minutes**	**165 minutes**

Table S2. The following table summarizes the validation metric values for different databases and sizes. The average values were obtained by generating all possible visualizations with MAYA. For each database, 56 chemical spaces were constructed.

Database (size)	Mean of the metrics
DNMT1 Inhibitors (471 compounds)	Trustworthiness: 0.914 ± 0.073<br>Correlation: 0.630 ± 0.164
Database of natural product BIOFACQUIM (532 compounds)	Trustworthiness: 0.903 ± 0.092<br>Correlation: 0.626 ± 0.121
G9A inhibitors from ChEMBL (428 compounds)	Trustworthiness: 0.925 ± 0.066<br>Correlation: 0.674 ± 0.227
Multi-target compounds for AR and PTP1B (813 compounds)	Trustworthiness: 0.927 ± 0.074<br>Correlation: 0.719 ± 0.102

Figure S1. Descriptive analysis of the properties of the 2309 FDA approved compounds**.** The analysis reveals that the

compounds exhibit similar values for molecular weight (MW), number of hydrogen bond acceptors (HBA), number of hydrogen bond donors (HBD), octanol/water partition coefficient (LogP), total topological surface area (TPSA), and number of rotatable bonds (RotBonds), as indicate by the large frequency bars in each graph.

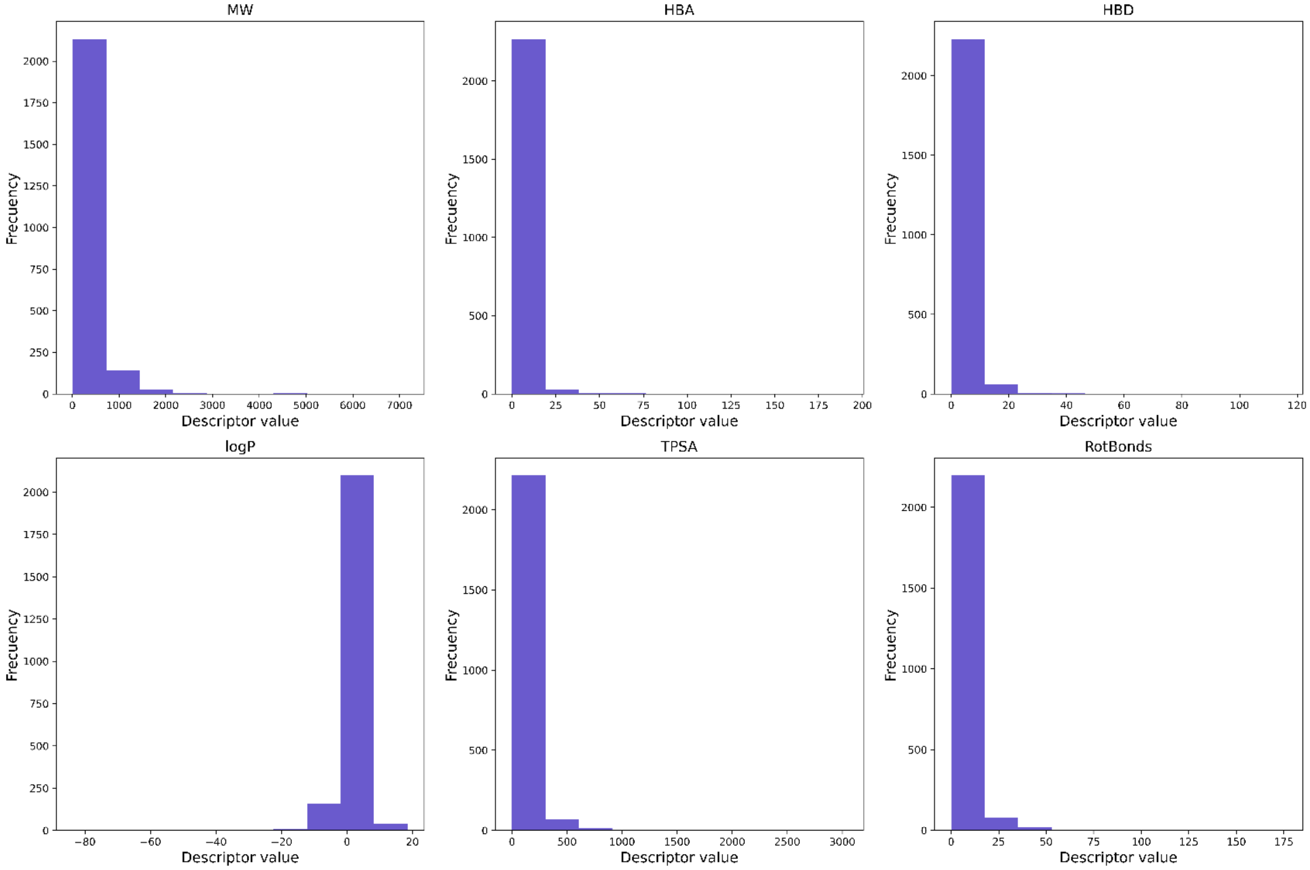

Figure S2. Visualizations of the chemical multiverse of FDA approved compounds obtained with MAYA. Due to the high

similarity among the compounds, data clustering is expected and is observed across all chemical spaces. MAYA demonstrates its ability to generate plots that preserve the local relationships of compounds, archiving Trustworthiness values greater than 0.9 for most visualizations and correlations above 0.6. This suggests that structurally similarity compounds remains close in the reduced dimensional space. The analysis was performance of perplexity=40, 1000 iterations, repeated in triplicate, yielding consistent results.

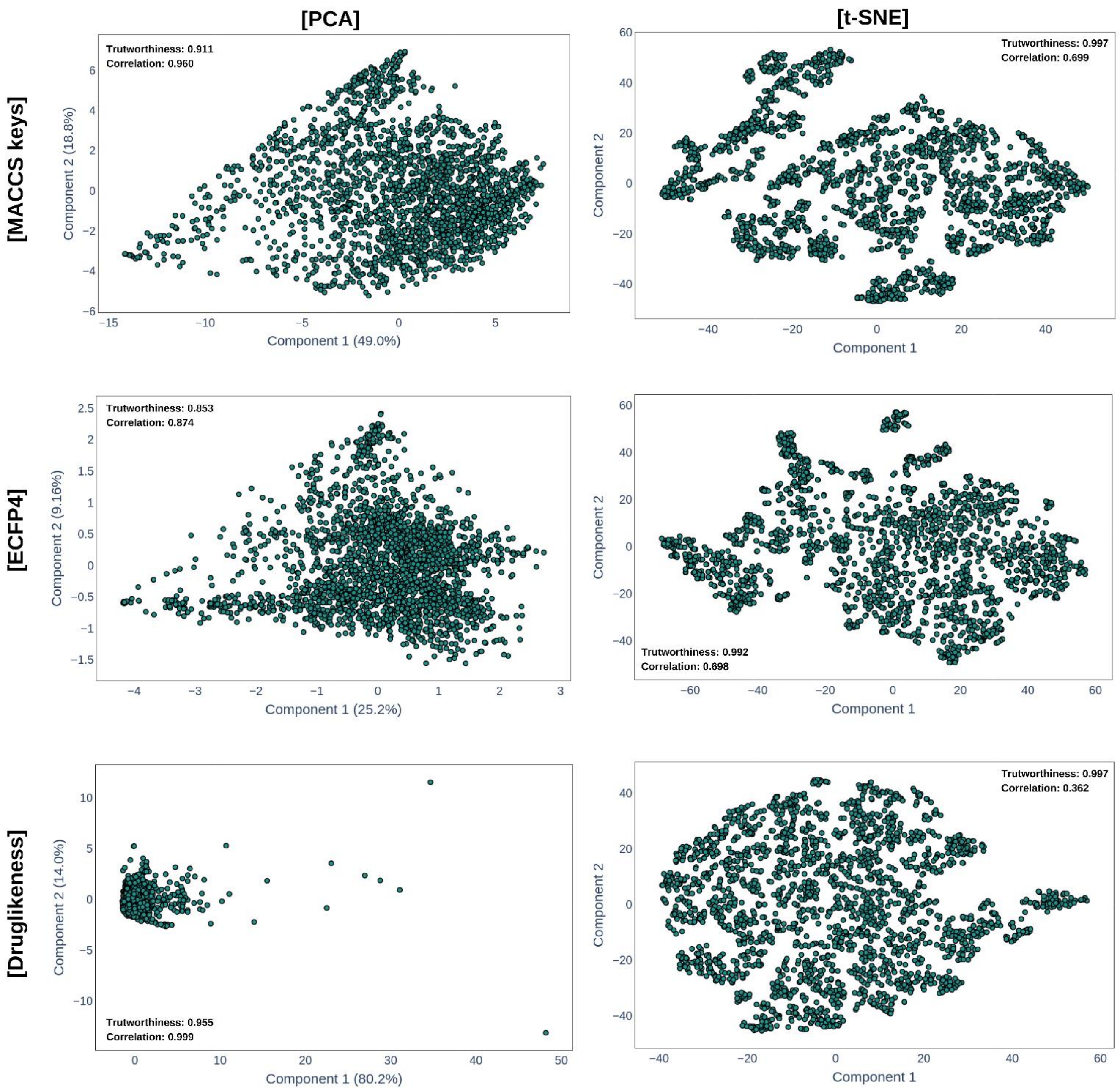

Figure S3. Chemical space visualizations automatically generated with MAYA for a database of 1,492 compounds annotated

with pK_I values against 172 kinases. [a] Visualizations using MACCS keys, achieving a recovery of the original variability of 50.4% (Trustworthiness=0.860 and Correlation=0.886). [b] Chemical space constructed with t-SNE and MACCS keys (Trustworthiness=0.993 and Correlation=0.643). No relevant regions were observed due to the similar clustering of compounds with high and low promiscuity values. Additionally, some visualizations using bioactivity descriptors were integrated. [c] The space constructed with PCA and the descriptor related to the mechanism of action of the compounds showed a homogeneous dispersion of the data (Trustworthiness=0.730 and Correlation=0.463). [d] However, when the visualization technique was modified to t-SNE, two regions were observed where data points with high promiscuity values clustered with smaller point sizes, indicating higher activity (Trustworthiness=0.979 and Correlation=0.417). This suggests a region of compounds that act similarly in the organism, exhibiting reduced activity across the 172 kinases and lacking selectivity. [e] The scaffold-based chemical space constructed with PCA showed a low variability recovery (21.4%) (Trustworthiness=0.80 and Correlation=0.404).

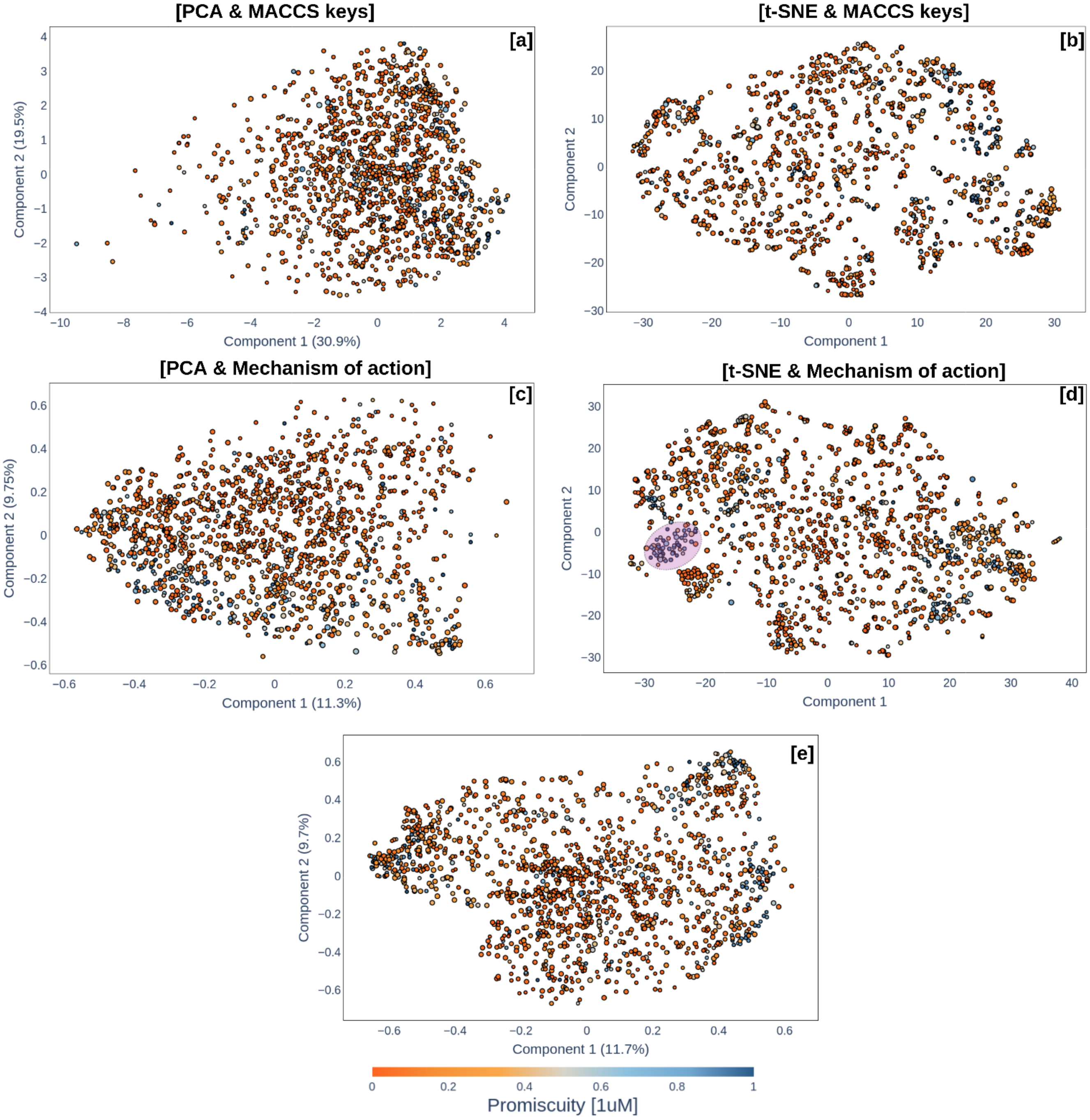

Figure S4. Herein we show the data visualization of an exemplary data set of 170 compounds annotated with inhibitory activity (IC50) against three biological endpoints, specifically with ChEMBL identifiers (IDs) CHEMBL3142, CHEMBL5147, and CHEMBL3234. The dataset was obtained from ChEMBL-34[28] focusing on small molecules with activity against kinase human enzymes, all evaluated with the same ChEMBL assay ID. Six of the eighteen possible visualizations generated with MAYA are shown. In this example, we show the PCA and t-SNE visualizations using three types of molecular representations (two types of molecular fingerprints and continuous properties of pharmaceutical relevance). The data points are coded on a continuous color scale from orange (compounds with overall low biological activity against the three biological endpoints) to blue (compounds with higher biological activity for all three kinases). The size of the data points represents the relative standard deviation of the biological activities (e.g., smaller data points are associated with molecules with lower standard deviation).

The modification of the employed descriptor and the visualization technique result in changes in data dispersion. This occurs because each descriptor captures distinct structural information, leading to the formation of different local clusters corresponding to similar compounds based on the features encoded by the descriptors. Figure S3 shows a clear difference in the arrangement of the dataset, it is observed when modifying the visualization technique and the molecular representation, resulting in distinct local clusters within the chemical space shared. Analysis of the visual representation of the chemical space is based on the similarity principle, compounds with similar properties (e.g., biological activity) share similar structural characteristics. Consequently, the formation of several local clusters, combined with the activity observed for each molecular target, enable a deeper level of analysis.

This analysis suggests that a higher density of points in regions with a lower mpIC50 value correlates with a greater contribution of that property to the reported experimental activity. Therefore, since active compounds share the same chemical space, the development of active compounds against the three targets can be guided by focusing on compounds within these regions. For example the chemical spaces constructed with the ECPF-6 descriptor show a greater clustering of the data. However, no clear separation is observed between active and inactive compounds for the three targets. This suggest that, at least for the compounds in the database, structural similarity is not a determining factor for activity.

However, in regions where a mixture of points of varying sizes is observed, these correspond to areas of the chemical space containing compounds with high variability in their activity against the three targets. Smaller points represent lower standard deviation, while larger points indicate greater differences in activity across the three targets. These larger points also display different mpIC50 values, corresponding to regions where it is not possible to establish structure-activity relationships.
This limitation arises because the descriptor used is insufficient to capture the features associated with the activities, failing to cluster compounds with similar mpIC50 values. Since compounds similar with respect to the same type of descriptor are expected to exhibit similar activity, the observed lack of clustering indicates a deficiency in the descriptor’s ability to encode the key features. Observing a greater degree of dispersion in other plots further supports the conclusion that the descriptor cannot encode the key property responsible for distinguishing more active compounds.

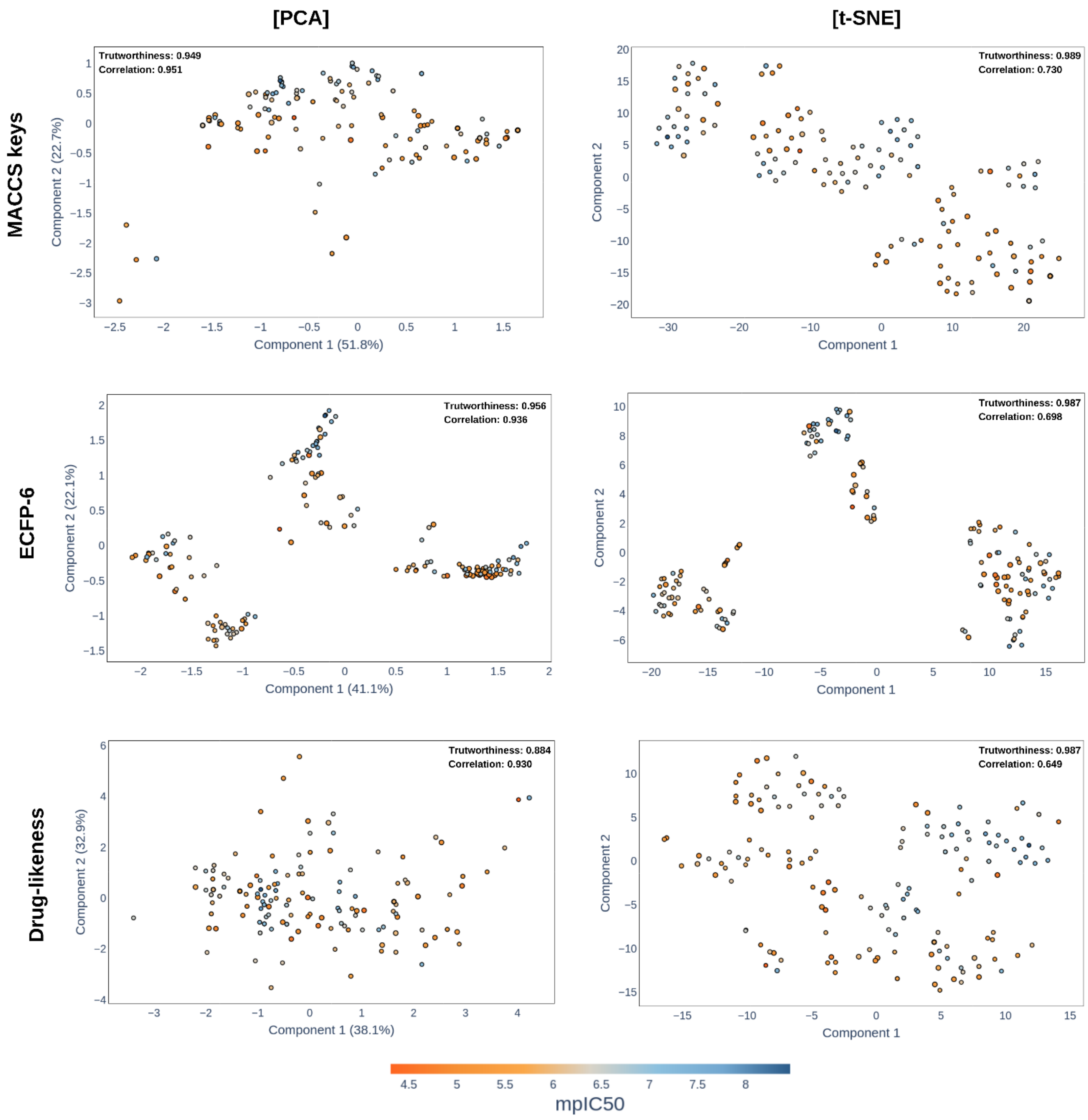
[PCA]
[t-SNE]
MACCS keys
ECFP-6
Drug-likeness
Trutworthiness: 0.949
Correlation: 0.951
Trutworthiness: 0.989
Correlation: 0.730
Trutworthiness: 0.956
Correlation: 0.936
Trutworthiness: 0.987
Correlation: 0.698
Trutworthiness: 0.884
Correlation: 0.930
Trutworthiness: 0.987
Correlation: 0.649
Component 1 (51.8%)
Component 2 (22.7%)
Component 1 (41.1%)
Component 2 (22.1%)
Component 1 (38.1%)
Component 2 (32.9%)
Component 1
Component 2
mpIC50

Figure S5. Visual representation of the chemical multiverse obtained automatically with MAYA using biological descriptors. A1) Scaffolds, C1) Small molecule roles. Overall, we observe a higher degree of data set dispersion. The biological descriptor C1 shows a better separation between active and inactive compounds.

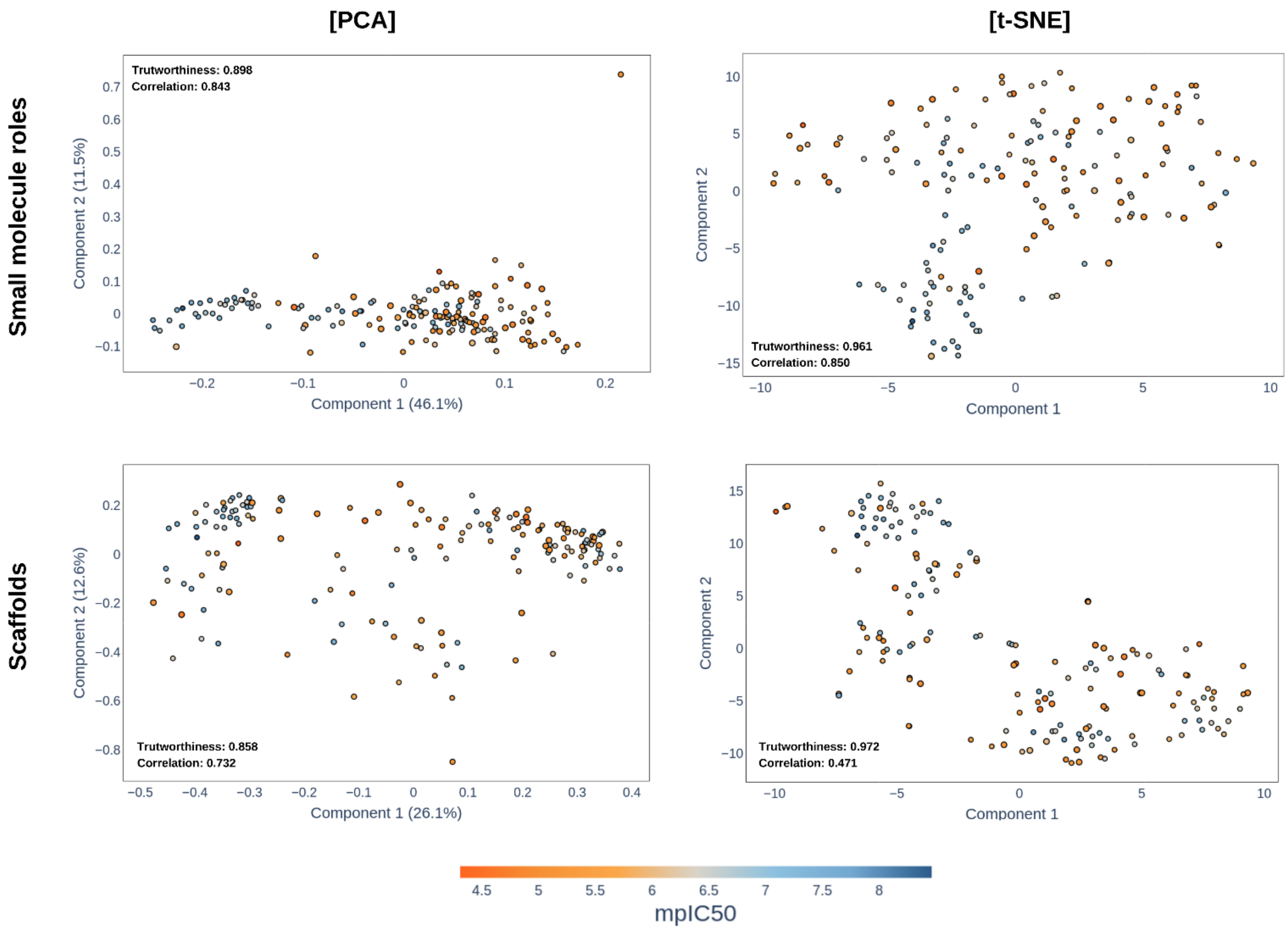

Supplement: Supplementary file 1 — Supporting Information [file MINF-44-e202400306-s001.pdf]
